# Identification of CD318 (CDCP1) as novel prognostic marker in AML

**DOI:** 10.1007/s00277-020-03907-9

**Published:** 2020-01-21

**Authors:** Jonas S. Heitmann, Ilona Hagelstein, Clemens Hinterleitner, Malte Roerden, Gundram Jung, Helmut R. Salih, Melanie Märklin, Joseph Kauer

**Affiliations:** 1 Clinical Collaboration Unit Translational Immunology, German Cancer Consortium (DKTK), Department of Internal Medicine, University Hospital Tübingen, Tübingen, Germany; 2 DFG Cluster of Excellence 2180 ‘Image-guided and Functional Instructed Tumor Therapy’ (IFIT), University of Tübingen, Tübingen, Germany; 3 Department of Internal Medicine VIII, University Hospital Tübingen, Tübingen, Germany; 4 University Hospital Tübingen, Department of Oncology, Haematology and Immunology, Tübingen, Germany; 5 Department of Immunology, German Cancer Consortium (DKTK) and German Cancer Research Center (DKFZ), Interfaculty Institute for Cell Biology, University of Tübingen, Partner Site Tübingen, Tübingen, Germany

**Keywords:** AML, CD318, CDCP1, Prognosis, Risk stratification

## Abstract

Genetic and morphological markers are well-established prognostic factors in acute myeloid leukemia (AML). However, further reliable markers are urgently needed to improve risk stratification in AML. CD318 (CDCP1) is a transmembrane protein which in solid tumors promotes formation of metastasis and correlates with poor survival. Despite its broad expression on hematological precursor cells, its prognostic significance in hematological malignancies so far remains unclear. Here, we evaluated the role of CD318 as novel prognostic marker in AML by immunophenotyping of leukemic blasts. Flow cytometric evaluation of CD318 on leukemic cells in 70 AML patients revealed a substantial expression in 40/70 (57%) of all cases. CD318 surface levels were significantly correlated with overall survival in patients receiving anthracycline-based induction therapy or best available alternative therapy. Using receiver-operating characteristics, we established a cut-off value to define CD318^lo^ and CD318^hi^ expression in both cohorts. Notably, high CD318 expression correlated inversely as prognostic marker in both treatment cohorts: as poor prognostic marker in patients receiving intense therapy, whereas upon palliative care it correlated with better outcome. In conclusion, FACS-based determination of CD318 expression may serve as novel prognostic factor depending on implemented therapy in AML patients.

**Electronic supplementary material:**

The online version of this article (10.1007/s00277-020-03907-9) contains supplementary material, which is available to authorized users.

## Introduction

Acute myeloid leukemia (AML) is a disease with high mortality and variable prognosis [1, 2]. Well-established prognostic factors are cytogenetic aberrations such as t(8;21), inv(16), and t(15;17) as well as mutated IDH1/2, NPM1, and FLT3 genes [3]. Immunophenotyping via flow cytometry comprises an additional fast technique to predict outcome in AML, although only few markers are yet established as prognostic factors in clinical routine diagnosis, despite the fact that new and rapidly available markers are needed to improve the treatment decisions in AML patients. This is even more since therapy in AML patients must be initiated immediately after diagnosis [1].

CD318 (CUB domain containing protein-1, CDCP1) is a highly glycosylated single pass transmembrane protein expressed on mesenchymal and neural stem cells and fibroblasts and hematopoietic progenitors [4–7]. CD318 overexpression has been recently correlated with poor overall survival (OS) in colon, breast, lung, renal, hepatocellular, and pancreatic carcinoma [8–14], possibly due to its role in metastasis formation via interaction with integrins and anti-apoptotic signaling via Akt [15–18].

In hematopoietic cells, CD318 has been identified as stem cell marker for both benign and malignant progenitor cells. CD318^+^ bone marrow or cord blood-derived cells have been shown to be capable to initiate multi-lineage hematopoiesis in vitro and in vivo [6]. Of AML blasts, pronounced CD318 expression has been observed on the immature CD34^+^CD133^+^ leukemic cells subset implicated to be enriched for leukemic stem cells [5, 6]. However, the impact of CD318 on survival in hematological malignancies has so far not been analyzed.

In this study, we investigated the role of flow cytometric analysis of AML blasts (*n* = 70) and correlated CD318 expression with disease outcome.

## Materials & methods

### Patient samples

Peripheral blood samples of 70 patients with AML were drawn at primary diagnosis. Peripheral blood mononuclear cells (PBMC) of patients were isolated by density gradient centrifugation and used for flow cytometry. Median observational time for all patients was 554 days (95% CI of mean 409–698 days). Diagnosis and classification of AML samples relied on morphology and cytochemistry of bone marrow according to the French-American-British (FAB) classification [19, 20]. Cytogenetic analyses were performed at the University of Ulm with standard methods.

### Flow cytometry

PBMC of AML patients were treated with human IgG (Sigma-Aldrich, St. Louis, MO) prior to the staining in order to minimize Fcɣ receptor binding, then washed and followed by adding the unconjugated CD318 mAb (clone CUB1, Biolegend, San Diego, CA) or the isotype control at 1 μg/ml, followed by species-specific PE-conjugated antibodies (1:100). Then, AML blasts were identified according to the immunophenotype obtained at diagnosis by staining for CD33, CD34, CD38, CD117, and CD13. Dead cells were excluded based on 7-AAD (BioLegend) positivity. Fluorescence-conjugates (CD13, CD33, CD34, CD38, and CD117, BioLegend) were used in 1:100-1:200 dilutions. Specific fluorescence indices (SFIs) were calculated by dividing median fluorescence obtained with anti-CD318 mAb by median fluorescence obtained with the IgG2b isotype control. Positive expression was defined as SFI ≥ 1.5. Measurements were conducted using a LSR Fortessa or a FACSCanto II (BD Biosciences, Heidelberg, Germany), and data analysis was performed with FlowJo_V10 software (FlowJo LCC, Ashland, OR). Graphs were created using GraphPad Prism 8.1.0 (GraphPad Software, San Diego, CA).

### Statistical analysis

Data are shown as mean ± SD and boxplots including mean and 25 and 75% quartiles as well as min/max or Tukey whiskers. To compare individual groups, the 2-tailed unpaired students *t* test, Mann-Whitney-/Kruskal-Wallis-test, Chi square test, or Fisher’s exact test were used. Distribution of overall survival (OS) was calculated using the Kaplan-Meier method. Log-rank test was performed to compare survival between groups. For predictive cut-off value estimation, we sub-grouped CD318 SFI with respect to corresponding OS times and by employed treatment. Receiver-operating characteristics (ROC) analysis was performed using JMP® Pro (SAS Institute Inc., Version 14.2), and value of highest Youden index was used as cut-off. Cut-off values enabled further separation of cases with better or worse prognosis, as shown in Kaplan-Meier analysis. Statistical analyses were conducted using JMP® Pro and GraphPad Prism 8.1.0 software. *P* values of < 0.05 were considered statistically significant.

## Results

### Clinical features of AML patients

For the analysis of CDCP1 expression, we analyzed primary AML samples of 70 patients. The clinical characteristics of the patients are given in Table 1 and Supplementary Table 1. Twenty patients presented with undifferentiated leukemia (M0: *n* = 6, M1: *n* = 14), 17 with immature granulocytic leukemia (M2: *n* = 14, M3: *n* = 5), and 21 with monocytic leukemia (M4: *n* = 12, M5: *n* = 9); nine patients had erythroleukemia (M6). In 52 patients, primary AML was diagnosed, and in 18 patients secondary AML was diagnosed. The age ranged from 21 to 85 years (with a median of 64 years) with a male:female ratio of 1:1.26. With regard to cytogenetics, 33 patients presented with normal karyotype, 24 with < 3 aberrations, and seven with complex karyotype. In detail, 5 cases with t(15;17), 3 with inv(16), as well as 3 with t(9;11) were included. FLT3-TKD mutations were detected in 6 patients, while a higher number (*n* = 25) presented with FLT3-ITD mutation, 15 of whom displayed a high FLT3-ITD ratio. NPM1 mutations were detected in 25 patients, while CCAAT/enhancer binding protein α (CEBPA) mutations were seen in 5 patients, and IDH2 mutations in 4 patients. Mutational status of TP53 was not assessed. On the basis of cytogenetics, patients were categorized according to National Comprehensive Cancer Network (NCCN) risk score [21]. Patient numbers in the favorable and intermediate risk group were 24, whereas 17 cases presented with adverse risk.

**Table 1:** Patients’ characteristics

	Number of patients (%)
Sex	
Male	31 (44)
Female	39 (56)
Median age (years)	64 (range 21–85)
FAB classification	
M0	6 (9)
M1	14 (20)
M2	14 (20)
M3	5 (7)
M4	12 (17)
M5	9 (13)
M6	9 (13)
Not classified	1 (1)
Unfavorable FAB	15 (22)
WHO classification	
AML with recurrent genetic abnormalities	37 (53)
AML with myelodysplasia-related changes	12 (17)
Therapy-related myeloid neoplasms	3 (4)
Myeloid neoplasms with germline predisposition	0 (0)
AML, not otherwise specified	18 (26)
Primary/secondary AML	
Primary	52 (74)
Secondary	18 (26)
Blood count	
WBC (G/L)	61 (range 5–448)
Hb (g/dl)	8 (range 4–13)
Plt (G/L)	41 (range 6–243)
NCCN risk score distribution	
Favorable	24 (34.5)
Intermediate	24 (34.5)
Poor	17 (24)
Not classified	5 (7)
Complete response after induction therapy ^π^	27 (66)

*FAB*, French-American-British; *WBC*, white blood count; *Hb*, hemoglobin; *Plt*, thrombocytes; *NCCN*, National Comprehensive Cancer Network

^π^only patients receiving anthracycline-based induction therapy, response assessment on day 25–35 after induction (CR, CRi)

Induction therapy was applied to 42 patients; the remaining patients (*n* = 28) were treated with other approved therapies (e.g., hydroxyurea, hypomethylating agents) or best supportive care. Response to chemotherapy was defined according to the European Leukemia Network (ELN) definition [22]. Complete response (CR) was defined as presentation with normocellular bone marrow containing < 5% blasts and neutrophilic granulocytes in peripheral blood (PB) recovered to 1500/μl and platelets to 100,000/μl. In contrast, complete remission with incomplete blood count recovery (CRi) lacked hematologic recovery in PB with neutrophil counts below 1000/μl or platelets below 100,000/μl. CR after anthracycline-based induction therapy was reached in 66% of treated patients.

### Prognostic evaluation of CD318 expression in AML

**Fig. 1:**
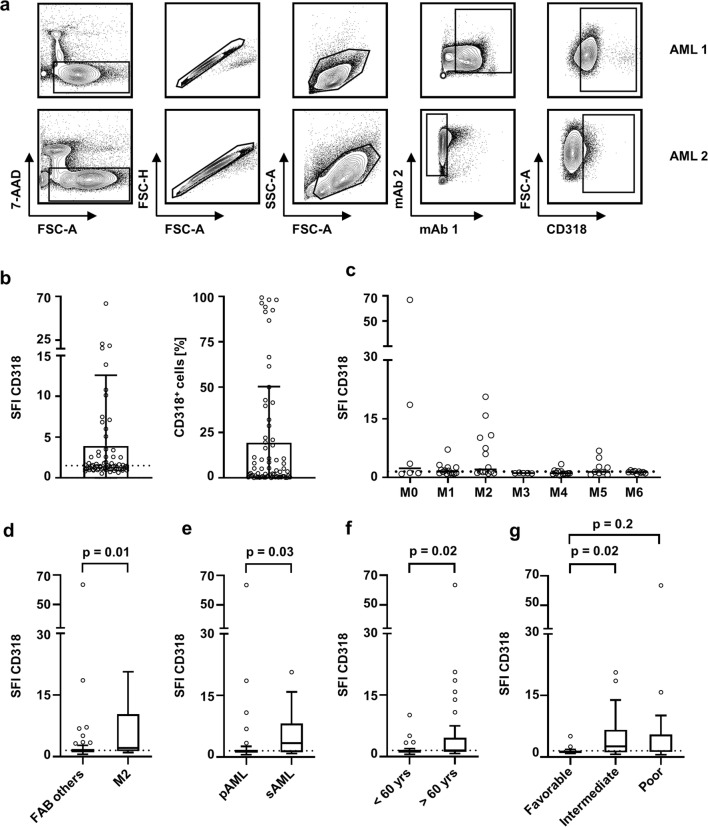
CD318 expression on hematopoietic cells and association with clinical parameters. CD318 expression was analyzed on hematopoietic cells by flow cytometry. SFI levels above 1.5 were considered as positive expression (dotted line). **a** Gating strategy for two exemplary AML samples: viable (7-AAD-), singlets, mononuclear cells, blast marker (AML 1: CD13/CD33, AML 2: CD33/CD14-), and CD318 expression. **b** CD318 expression on blasts of AML patients (*n* = 70) are depicted as SFI levels and percentage of CD318 positive blasts (boxplots with min/max whiskers). **c**–**e** CD318 SFI levels according to the different FAB classifications (single values, median) (**c**), FAB others vs. FAB M2 (boxplots with Tukey whiskers; Mann-Whitney test) (**d**), primary (pAML) vs. secondary (sAML) AML (boxplots with Tukey whiskers; Mann-Whitney test) (**e**), and according to age < 60 and > 60 years (boxplots with Tukey whiskers; Mann-Whitney test) (**f**) are shown. **g** Distribution of CD318 expression (SFI) throughout NCCN risk group (boxplots with Tukey whiskers; Kruskal-Wallis test)

Leukemic blasts were analyzed for CD318 expression by flow cytometry as exemplified in Fig. 1a. Considering an SFI level of 1.5 a as margin for positivity, 57% of AML patients expressed relevant CD318 levels. Among all AML cases, highly variable surface levels of CD318 ranging from 1.5 to SFI levels of 66.8 were observed. In addition, the percentage of positive cells varied substantially ranging from 0 to almost 100% of the leukemic blasts (Fig. 1b).

Distribution of CD318 surface levels differed among FAB subclasses, with a tendency to lower expression in mature leukemia (FAB M4/5) and higher expression in immature leukemia (Fig. 1c). In comparison to all other FAB subtypes (*n* = 56), cases with FAB M2 (*n* = 14) expressed the highest CD318 levels (*p* = 0.01) (Fig. 1d).

CD318 expression further varied between primary (pAML) and secondary AML (sAML) with a significantly higher expression in the latter (SFI mean 3.3 vs. 5.7; *p* = 0.025) (Fig. 1e). In line, classification using WHO criteria revealed a significantly higher CD318 SFI in “AML with myelodysplasia-related changes” compared with “AML with recurrent genetic abnormalities” (data not shown). Furthermore, on leukemic blasts of patients older than 60 years, CD318 expression was significantly enhanced in comparison to younger patients (*p* = 0.02) (Fig. 1f). When grouped according to the NCCN risk classification, patients with intermediate risk expressed higher levels of CD318 than favorable risk patients (*p* = 0.02), whereas this was not veritable in comparison to poor risk (*p* = 0.2) (Fig. 1g).

### Distinct CD318 expression profile on AML blasts of patients with poor prognosis

**Fig. 2:**
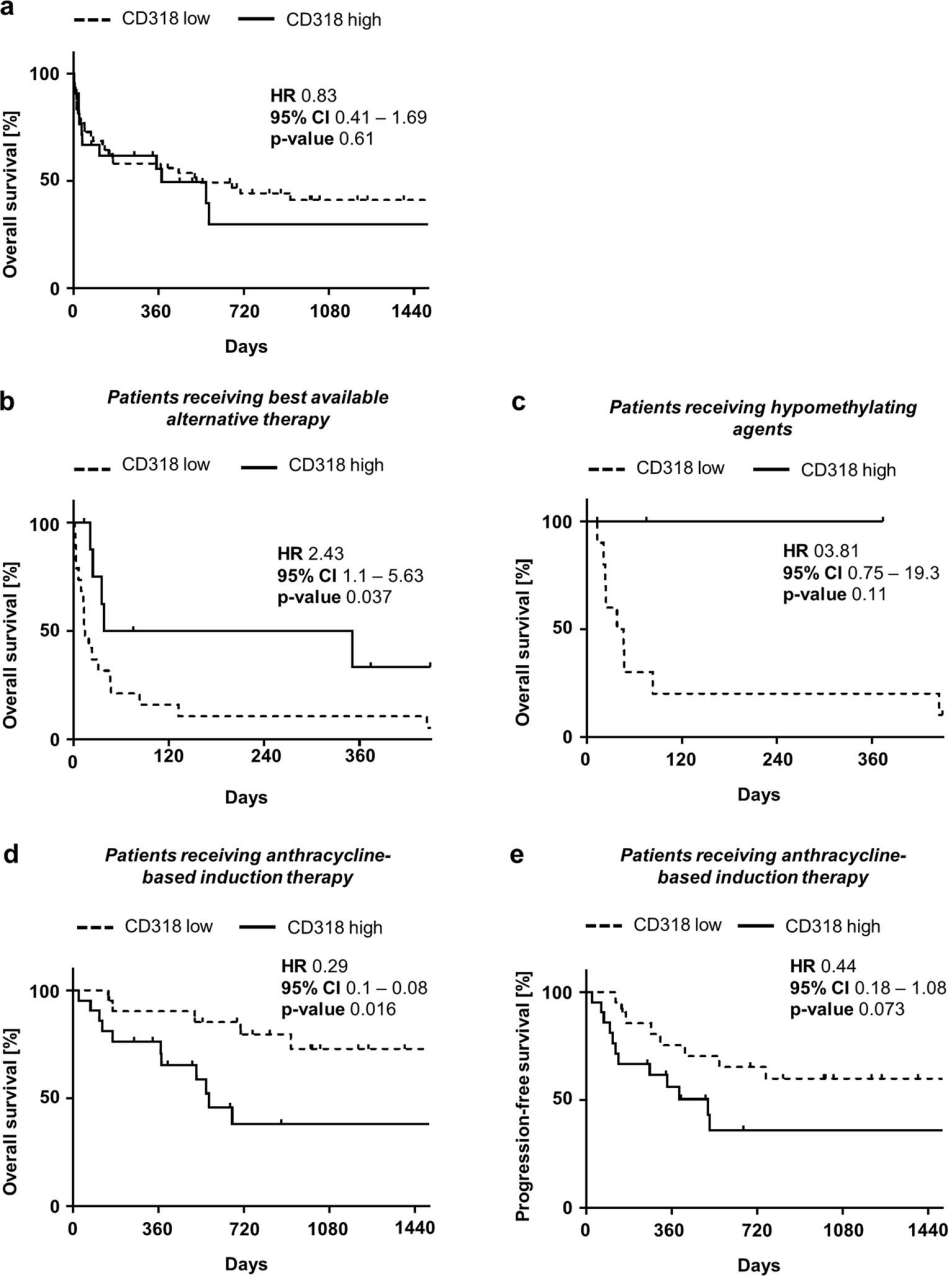
Impact of CD318 expression on clinical outcome. **a** Overall survival (OS) in AML patients according to CD318^lo^ and CD318^hi^ expression in Kaplan-Meier analysis. **b** Overall survival in patients without intense therapy according to CD318^lo^ and CD318^hi^ expression in Kaplan-Meier analysis. Mean OS was reached in CD318^lo^ (dotted line) and CD318^hi^ (continuous line) after 14 or 194.5 days, respectively, and differed significantly (log-rank test). **c** Overall survival in patients receiving hypomethylating agents according to CD318^lo^ and CD318^hi^ expression in Kaplan-Meier analysis. **d** Overall survival in patients receiving anthracyclin-based induction therapy (ABIT) according to CD318^lo^ and CD318^hi^ expression in Kaplan-Meier analysis. Mean OS was only reached in CD318^hi^ after 572 days (dotted line; log-rank test). **e** Progression-free survival (PFS) according to CD318^lo^ and CD318^hi^ expression in Kaplan-Meier analysis. In CD318^hi^, the mean PFS was 513 days (dotted line; log-rank test) and could not be reached in CD318^lo^ (continuous line)

Since CD318 expression varied among AML patient cells, for subsequent analysis, predicted cut-off values were estimated by ROC analysis. An SFI of 1.8 (AUC 0.56, 95% CI 0.42–0.7) separated all AML patients in CD318 high (CD318^hi^) and low (CD318^lo^) expressing cases. In our cohort including all therapy strategies, this estimated cut-off value and thereby grouping of AML patients did not reveal any difference on OS (hazard ratio (HR) 0.83; *p* = 0.61) (Fig. 2a). However, upon separating patients according to their applied therapy (anthracycline-based induction therapy vs. best available alternative treatment), a significant correlation of CD318 surface levels on AML blasts with prognosis was observed.

Considering clinical characteristics of patients, separation by a cut-off value of SFI 3.2 (AUC 0.82, 95% CI 0.6–1.0) for patients receiving best available alternative therapy identified a trend to more cases of secondary AML in CD318^hi^ patients, however without reaching statistical significance (*p* = 0.052). When patients were grouped according to NCCN risk score, we detected a significantly higher percentage of intermediate and poor risk patients in CD318^hi^ cases (*p* = 0.03). Interestingly, white blood count (WBC) was significantly increased in CD318^lo^ AML patients (*p* = 0.0006). Furthermore, the rate of NPM1 mutation was significantly higher in CD318^lo^ AML patients (*p* = 0.03) (Table 2 and Supplementary Table 2).

**Table 2:** Distribution of patients’ characteristics according to CD318^hi^ and CD318^lo^

	Best available alternative therapy			Anthracycline-based induction therapy		
	Number of patients (%)			Number of patients (%)		
	CD318^lo^ (SFI < 3.2) *n* = 19	CD318^hi^ (SFI ≥ 3.2) *n* = 9	*p* value	CD318^lo^ (SFI < 1.17) *n* = 21	CD318^hi^ (SFI ≥ 1.17) *n* = 21	*p* value
Sex						
Male	13 (68.5)	5 (55.5)	0.51^┘^	9 (43)	12 (57)	0.35^┘^
Female	6 (31.5)	4 (44.5)		12 (57)	9 (435)	
Median age (years)	73 (range 26–83)	78 (range 58–85)	0.15^†^	47 (range 23–73)	58 (range 21–74)	0.27^†^
FAB classification			0.19^‡^			0.15^‡^
M0	2 (10.5)	3 (37.5)		1 (5)	0 (0)	
M1	4 (21)	2 (25)		3 (14)	5 (24)	
M2	2 (10.5)	2 (25)		3 (14)	7 (33)	
M3	0 (0)	0 (0)		4 (19)	1 (4.5)	
M4	2 (10.5)	0 (0)		7 (33)	3 (14)	
M5	5 (26.5)	1 (12.5)		2 (9)	1 (4.5)	
M6	4 (21)	0 (0)		1 (5)	4 (20)	
Unfavorable FAB	6 (32.5)	3 (37.5)	0.77^┘^	2 (9.5)	4 (19)	0.37^┘^
WHO classification			0.13^‡^			0.68^‡^
AML with recurrent genetic abnormalities	9 (48)	1 (11)		15 (71)	12 (57)	
AML with myelodysplasia-related changes	5 (26)	3 (33.5)		1 (5)	3 (14)	
Therapy-related myeloid neoplasms	0 (7)	1 (11)		1 (5)	1 (5)	
Myeloid neoplasms with germline predisposition	0 (0)	0 (0)		0 (0)	0 (0)	
AML, not otherwise specified	5 (26)	4 (44.5)		4 (19)	5 (24)	
Primary/secondary AML						
Primary	14 (74)	5 (33)	0.052^┘^	19 (90.5)	16 (76)	0.21^┘^
Secondary	3 (26)	6 (67)		2 (9.5)	5 (24)	
Blood count						
WBC (G/L)	137.4	32.5	0.0006^†^	79.9	112.7	0.25^†^
Hb (g/dl)	8.6	8.8	0.69^†^	8.2	8.2	0.98^†^
Plt (G/L)	66.7	66.2	0.98^†^	51.5	75.7	0.15^†^
NCCN risk score distribution			0.03^‡^			0.28^‡^
Favorable	7 (37)	0 (0)		11 (52.5)	6 (29)	
Intermediate	6 (31.5)	6 (67)		5 (24)	7 (33)	
Poor	4 (21)	3 (33)		3 (14)	7 (33)	
Not classified	2 (10.5)	0 (0)		2 (9.5)	1 (5)	
Complete response after induction therapy^π^	n/a	n/a	n/a	15 (75)	12 (57)	0.23^┘^

*FAB*, French-American-British; *WBC*, white blood count; *Hb*, hemoglobin; *Plt*, thrombocytes; *NCCN*, National Comprehensive Cancer Network

^π^only patients receiving anthracycline-based induction therapy, response assessment: on day 25–35 after induction (CR, CRi). Statistical analysis with ^┘^Fisher’s exact test, ^‡^Pearson-Chi square and ^†^students *t* test

In CD318^lo^ patients receiving alternative therapy, OS was significantly worse when compared with CD318^hi^ cases (HR 2.43; *p* = 0.037) (Fig. 2a). These findings were supported by analysis of the subgroup of AML patients receiving hypomethylating agents (*n* = 13), which showed a clear trend to better OS in CD318^hi^ cases, however without reaching statistical significance (HR 3.81; *p* = 0.11) (Fig. 2c).

Subsequently, a cut-off value was estimated using ROC analysis in patients receiving anthracycline-based induction therapy (SFI 1.17, AUC 0.63, 95% CI 0.45–0.81). In this patient group, comparison of CD318^lo^ and CD318^hi^ cases revealed no statistical significance for any clinical parameter except for a higher rate of IDH2 mutations in CD318^hi^ cases (*p* = 0.04) (Table 2 and Supplementary Table 2).

However, CD318^hi^ cases receiving anthracycline-based induction therapy displayed a significantly lower OS in comparison to CD318^lo^ (HR 0.29; *p* = 0.016) (Fig. 2d). A similar trend was observed for progression-free survival (PFS), however without reaching statistical significance in our cohort (HR 0.44; *p* = 0.073) (Fig. 2e).

**Fig. 3:**
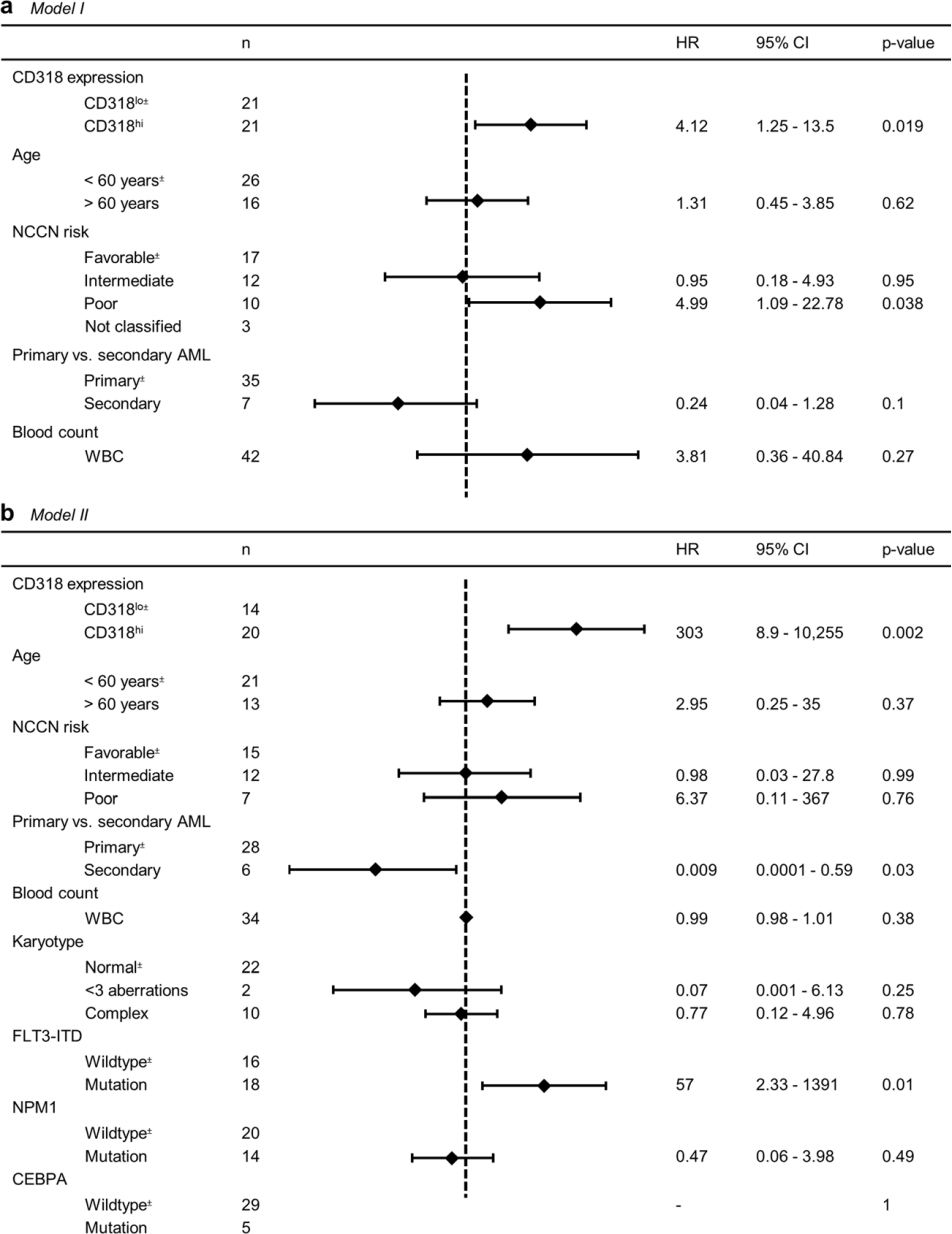
Multivariate analysis for survival in patients receiving anthracycline-based induction therapy. **a** Model I: all patients receiving anthracycline-based induction therapy (*n* = 42). **b** Model II: patients receiving anthracycline-based induction therapy with known cytogenetic parameters (*n* = 34). NCCN National Comprehensive Cancer Network; WBC white blood count; ^±^reference group, dotted line: HR = 1

To confirm these results in patients receiving anthracycline-based induction therapy, multivariate analysis including age (< 60 vs. ≥ 60 years), WBC, primary/secondary AML, risk profile according NCCN, and CD318 expression was conducted. A HR of 4.11 was calculated for CD318^hi^ which attributes the strongest correlation of all markers to CD318 expression (*p* = 0.02). As expected, poor NCCN risk cases showed a significantly decreased OS (*p* = 0.03). All other parameters showed no significant impact on OS (Fig. 3a).

In an alternative approach (depicted as model II), an extended multivariate analysis for patients receiving anthracycline-based induction therapy including age (< 60 vs. ≥60 years), WBC, primary/ secondary AML, risk profile according NCCN, karyotype, FLT3-ITD mutation status, NPM1 mutation status, CEBPA mutation status, and CD318 expression was performed. Eight patients were excluded due to unavailability of the cytogenetic parameters. A HR of 303 for CD318^hi^ underlines the strong correlation between CD318^hi^ expression and poor survival (*p* = 0.002). FLT3-ITD positive cases showed significantly decreased OS (*p* = 0.01), which is in line with the literature [1]. Cases of secondary AML showed increased OS (*p* = 0.03). All other parameters showed no significant impact on OS (Fig. 3b).

## Discussion

In this study, we analyzed CD318 expression on peripheral blasts of 70 AML patients and observed substantial CD318 expression in 57% of all patients. So far, only few publications have reported on the expression of CD318 in hematological malignancies [5, 6]. CD318 mRNA as well as surface expression was detected on the K562 cell line, but not in Jurkat (T cell leukemia) and Raji (Burkitt lymphoma) cell lines [23]. Bühring et al. implemented flow cytometry using the antibody CUB1 and showed surface expression on 7/11 AML patients [5]. The observed expression of CD318 in 57% of the analyzed AML cases in our cohort was comparable to that reported by Bühring et al.

Beside expression in various cancer entities, functionally relevant expression of CD318 was described on hematopoietic, neural, and mesenchymal stem cells [5, 6]. With regard to hematopoiesis, CD34^+^CD38^−^CD318^+^ cells comprise a group of myeloid progenitor cells [5], and CD318^+^ bone marrow- or cord blood-derived cells reportedly are particularly capable of forming multi-lineage hematopoiesis in vitro as well as in a xenograft model [6, 24]. In contrast to hematopoietic progenitor cells, the molecule is absent on mature lymphocytes, monocytes, granulocytes, erythrocytes, and platelets [6]. This suggests a role of CD318 as marker of myeloid progenitors in both benign and malignant hematological cells. Interestingly, our findings point in a similar direction, since undifferentiated subtypes of AML showed a higher CD318 expression in comparison to differentiated leukemic blasts.

CD318 expression has also been associated with the presence of genetic aberrations. For example, in lung carcinoma, CD318 surface levels correlate with Ras mutations [18]. In our study analyzing patients with AML, no association between genetic aberrations or mutations was observed, although we detected higher CD318 expression in secondary AML and patients with age above 60 years.

Besides other mechanisms, CD318 expression is induced via hypoxia-inducible factor (HIF)-2 alpha [25]. HIF-2 alpha protects hematopoietic progenitors as well as AML cells from apoptosis due to endoplasmic reticulum stress [26]. Moreover, it promotes AML progression in mouse models, but HIF-2 alpha is not established as prognostic marker in AML [27]. Further studies demonstrated an accelerated growth of CD318-transfected MCF-7 cells in mice compared with mock-transfected controls [28]. In addition, CD318 surface overexpression is correlated with poor overall survival in colon, breast, lung, renal, hepatocellular, and pancreatic carcinoma [8–14]. This has been attributed to the enhanced formation of metastasis via CD318 by its interaction with activated integrin β-1 during stromal invasion and transendothelial migration [15]. Metastasis promotion by CD318 includes cleavage of the membrane-bound protein by serine proteases and subsequent phosphorylation by Src kinases [16]. Finally, Akt activation inhibits PARP1-mediated apoptosis and promotes survival of tumor cells during extravasation and tissue invasion [17]. In addition, achievement of CR after intense therapy was not significantly different between CD318^lo^ and CD318^hi^ patients, as one would expect due to anti-apoptotic signaling via CD318.

Moreover, we were not able to detect a significant difference between CD318^lo^ and CD318^hi^ AML cases with regard to OS when we did not discriminate with regard to the applied treatment strategy. However, when we grouped our patients according to the applied treatment, we found that patients receiving intense AML treatment and expressing low levels of CD318^lo^ on AML blasts had significantly longer OS and a tendency to longer PFS. On the contrary, CD318^hi^ cases receiving best available alternative therapy showed increased survival.

Upon more detailed analysis, we identified 13 patients receiving hypomethylating agents as initial AML treatment. In this small group, there was a trend towards better OS with CD318^hi^ expression. Of note, CD318 expression reportedly is highly variable in other tumors and depends on DNA methylation of its promotor region [23, 29]. It has been reported that AML blasts are dependent on promoter methylation of tumor suppressor genes [30], which is inhibited by hypomethylating agents (e.g., decitabine) [31]. It is tempting to speculate that high expression of CD318 could be used as marker for the degree of DNA methylation in AML, and this issue should be addressed in future studies, as we were not able to analyze changes of CD318 surface expression upon treatment due to lack of respective patient samples.

If CD318 expression would be stable upon therapy, the CD318^hi^ AML patient population would potentially benefit from a combinational treatment with an anti-CD318 antibody, such as RG7287 (humanized CD318 antibody). This is based on observations that in vivo xenograft testing with a breast cancer cell line showed therapeutic potential of this antibody in combination with paclitaxel, with inhibition of metastasis being the most impressive read out [32].

In conclusion, to our knowledge, this is the first analysis of CD318 surface levels on leukemic cells in a larger cohort of AML patients. The observed association of CD318 expression with OS in patients receiving anthracycline-based induction therapy or best available alternative therapy points the suitability of CD318 analysis as prognostic marker and indicates that CD318 might serve as target antigen for immunotherapeutic approaches.

## Electronic supplementary material

ESM 1 (PDF 438 kb)
