# Supplementary material for: Identification of CD318 (CDCP1) as novel prognostic marker in AML
Source: Ann Hematol. 2020 Jan 21;99(3):477–86. doi: 10.1007/s00277-020-03907-9 (PMC7060168; doi:10.1007/s00277-020-03907-9)
Supplement: Supplementary file 1 — (PDF 438 kb) [file 277_2020_3907_MOESM1_ESM.pdf]

Supplementary Information

Article in *Annals of Hematology*

Identification of CD318 (CDCP1) as novel prognostic marker in AML

Jonas S. Heitmann, Ilona Hagelstein, Clemens Hinterleitner, Malte Roerden, Gundram Jung, Helmut R. Salih, Melanie Märklin, Joseph Kauer

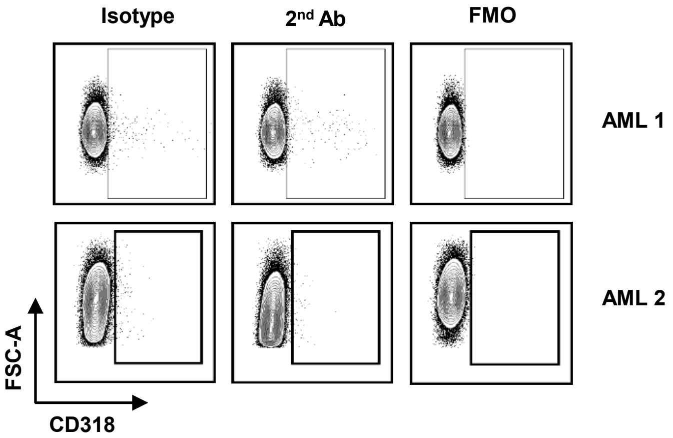

Supplementary Fig. 1 Flow cytometry controls

CD318 expression was analyzed on hematopoietic cells by flow cytometry. Controls for two exemplary AML samples are depicted: Flourescence-minus-one control (FMO), secondary antibody-only and IgG2b isotype control.

Supplementary Table 1 Patients cytogenetic characteristics

	Number of patients (%)
Karyotype	
Normal	33 (47)
<3 aberrations	24 (34)
Complex karyotype	7 (10)
Not determined	6 (9)
t(15;17); PML-RARA	
Negative	63 (90)
Positive	5 (7)
Not determined	2 (3)
t(8;21); RUNX1-RUNX1T1	
Negative	66 (94)
Positive	0 (0)
Not determined	4 (6)
inv(16); CBFB-MYH11	
Negative	64 (92)
Positive	3 (4)
Not determined	3 (4)
t(9;11); MLL-AF9	
Negative	56 (80)
Positive	3 (4)
Not determined	11 (16)
FLT3-ITD	
Wildtype	40 (57)
Mutation	25 (36)
Not determined	5 (7)
FLT3-ITD ratio	
High	15 (21)
Low	10 (14)
Not determined	45 (65)
FLT3-TKD	
Wildtype	59 (84)
Mutation	6 (9)
Not determined	5 (7)
NPM1	
Wildtype	40 (57)
Mutation	25 (36)
Not determined	5 (7)
CEBPA	
Wildtype	54 (77)
Mutation	5 (7)
Not determined	11 (16)
IDH	
Wildtype	21 (30)
Mutated IDH1	0 (0)
Mutated IDH2	4 (6)
Not determined	45 (64)

Supplementary Table 2 Distribution of patients cytogenetic characteristics according to $CD318^{hi}$ and $CD318^{lo}$

	Best available alternative therapy			*Anthracycline-based induction therapy*		
	Number of patients (%)			Number of patients (%)		
	$CD318^{lo}$ (SFI < 3.2) n =19	$CD318^{hi}$ (SFI ≥ 3.2) n =9	p-value	$CD318^{lo}$ (SFI < 1.17) n =21	$CD318^{hi}$ (SFI ≥ 1.17) n =21	p-value
Karyotype			0.33‡			0.83‡
Normal	11 (58)	2 (22)		9 (43)	11 (52)	
<3 aberrations	4 (21)	4 (45)		8 (38)	8 (38)	
Complex	2 (10.5)	2 (22)		2 (9.5)	1 (5)	
Not determined	2 (10.5)	1 (11)		2 (9.5)	1 (5)	
t(15;17); PML-RARA			1┘			
Negative	17 (89.5)	9 (100)		17 (81)	20 (95)	0.17┘
Positive	0 (0)	0 (0)		4 (19)	1 (5)	
Not determined	2 (10.5)	0 (0)		0 (0)	0 (0)	
t(8;21); RUNX1-RUNX1T1			1┘			0.76┘
Negative	17 (89.5)	9 (100)		20 (95)	20 (95)	
Positive	0 (0)	0 (0)		0 (0)	0 (0)	
Not determined	2 (10.5)	0 (0)		1 (5)	1 (5)	
inv(16); CBFB-MYH11			1┘			0.48┘
Negative	17 (89.5)	9 (100)		18 (85)	20 (95)	
Positive	0 (0)	0 (0)		2 (10)	1 (5)	
Not determined	2 (10.5)	0 (0)		1 (5)	0 (0)	
t(9;11); MLL-AF9			0.39┘			0.59┘
Negative	12 (63)	8 (89)		17 (81)	19 (90)	
Positive	2 (10.5)	0 (0)		0 (0)	1 (5)	
Not determined	5 (26.5)	1 (11)		4 (19)	1 (5)	
FLT3-ITD			0.63┘			0.17┘
Wildtype	12 (63)	7 (78)		12 (57)	9 (43)	
Mutation	4 (21)	2 (22)		7 (33)	12 (57)	
Not determined	3 (16)	0 (0)		2 (10)	0 (0)	
FLT3-ITD ratio			0.60┘			0.29┘
Low	1 (5)	1 (11)		4 (19)	4 (19)	
High	3 (16)	1 (11)		3 (14)	8 (38)	
Not determined	15 (79)	7 (78)		14 (67)	9 (43)	
FLT3-TKD			0.60┘			0.27┘
Wildtype	15 (79)	8 (89)		16 (76)	20 (95)	
Mutation	1 (5)	1 (11)		3 (14)	1 (5)	
Not determined	3 (16)	0 (0)		2 (10)	0 (0)	
NPM1			0.03┘			0.40┘
Wildtype	7 (37)	8 (89)		11 (52)	14 (67)	
Mutation	9 (47)	1 (11)		8 (38)	7 (33)	
Not determined	3 (16)	0 (0)		2 (10)	0 (0)	
CEBPA			1┘			0.61┘
Wildtype	14 (73.5)	9 (100)		14 (66)	17 (81)	
Mutation	0 (0)	0 (0)		2 (10)	3 (14)	
Not determined	5 (26.5)	0 (0)		5 (24)	1 (5)	
IDH			0.12‡			0.04‡
Wildtype	4 (21)	6 (67)		8 (38)	3 (14)	
Mutated IDH1	0 (0)	0 (0)		0 (0)	0 (0)	
Mutated IDH2	2 (10.5)	0 (0)		0 (0)	2 (10)	
Not determined	13 (68.5)	3 (33)		13 (62)	16 (76)	

Statistical analysis with ┘ Fisher's exact test or ‡Pearson-Chi^2 .
